# Supplementary material for: Targeting MUC1-C Suppresses Chronic Activation of Cytosolic Nucleotide Receptors and STING in Triple-Negative Breast Cancer
Source: Cancers (Basel). 2022 May 24;14(11):2580. doi: 10.3390/cancers14112580 (PMC9179855; doi:10.3390/cancers14112580)

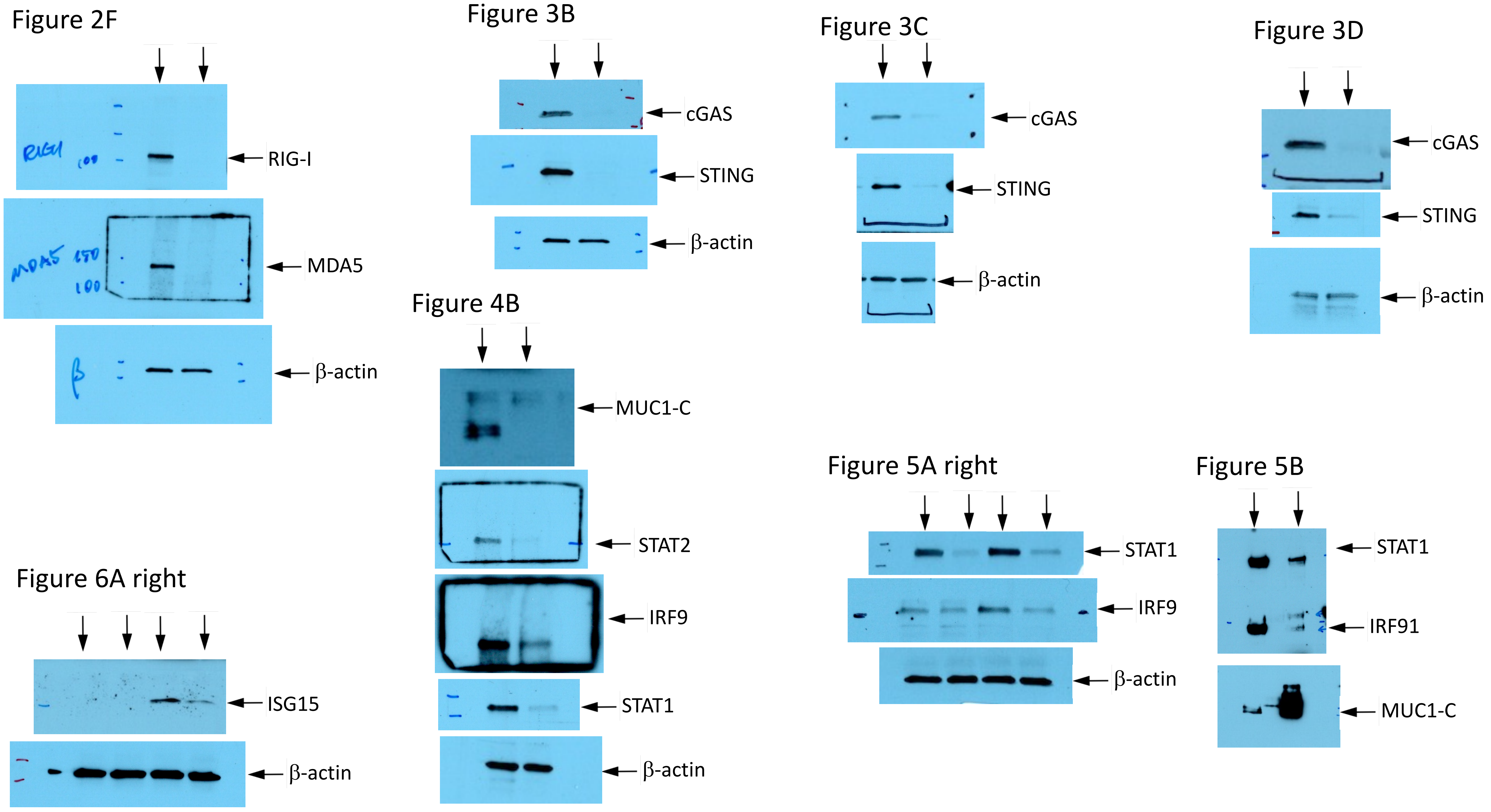
Figure 2F
RIG-I
MDA5
β-actin
Figure 3B
cGAS
STING
β-actin
Figure 3C
cGAS
STING
β-actin
Figure 3D
cGAS
STING
β-actin
Figure 4B
MUC1-C
STAT2
IRF9
STAT1
β-actin
Figure 5A right
STAT1
IRF9
β-actin
Figure 5B
STAT1
IRF91
MUC1-C
Figure 6A right
ISG15
β-actin

Figure 6D

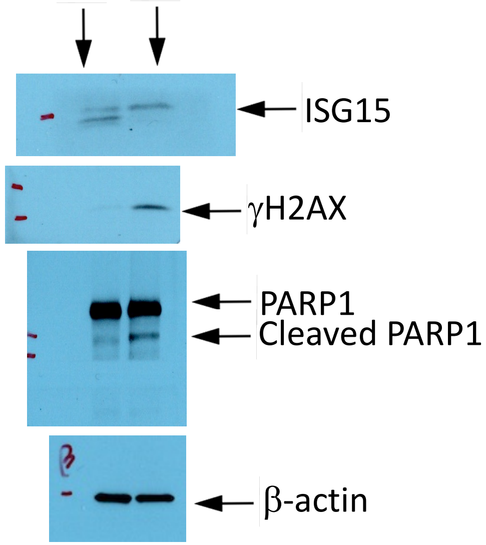

Figure 6E

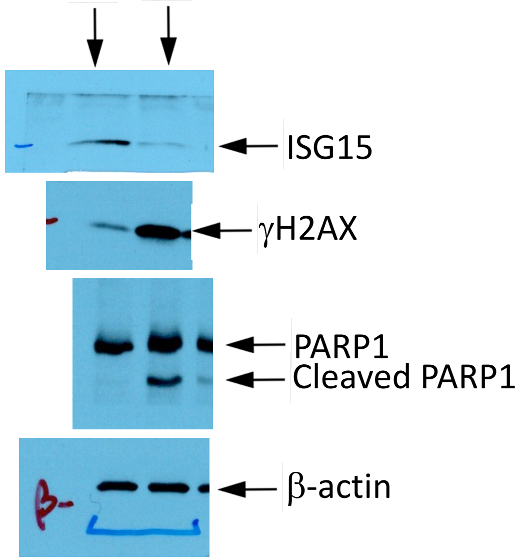

Figure 7A

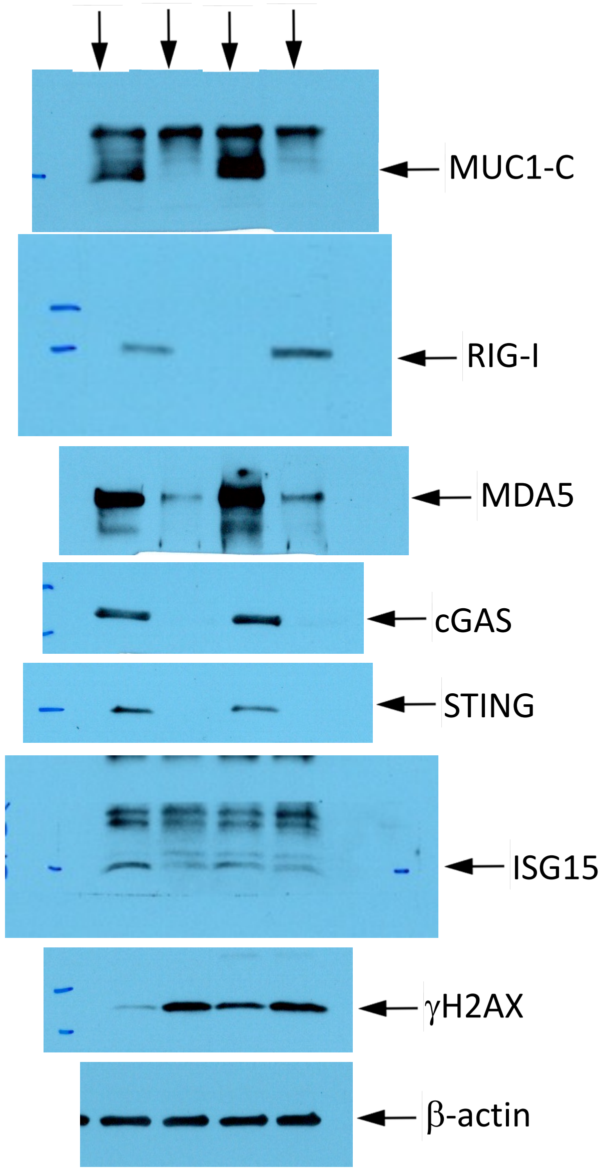

Figure 7B

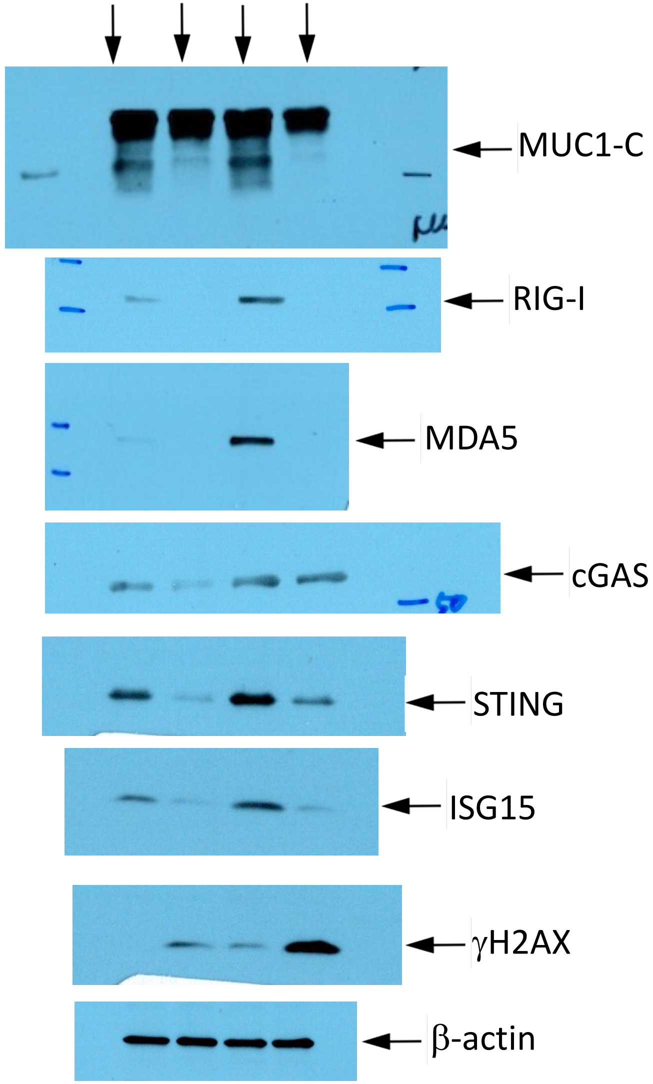

Figure S2A

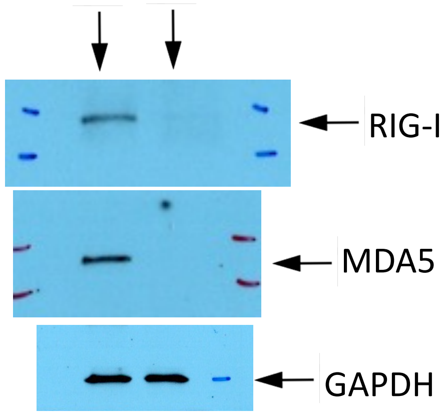

Figure S2B

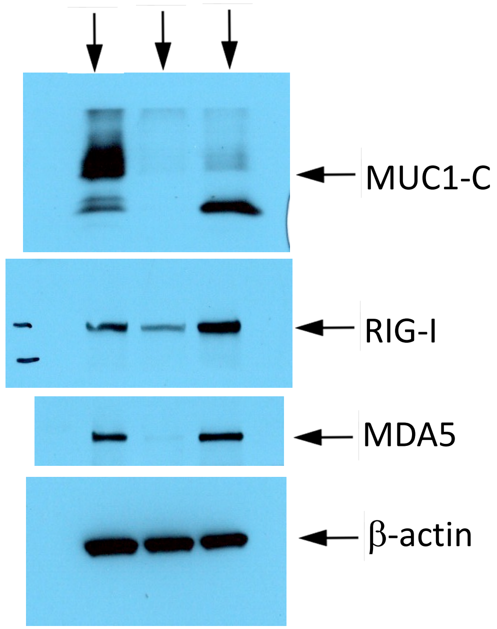

Figure S2D

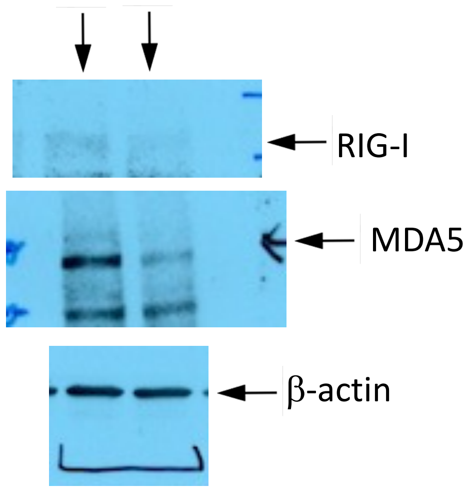

Figure S3C

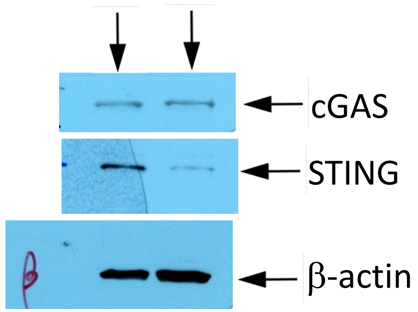

Figure S3D

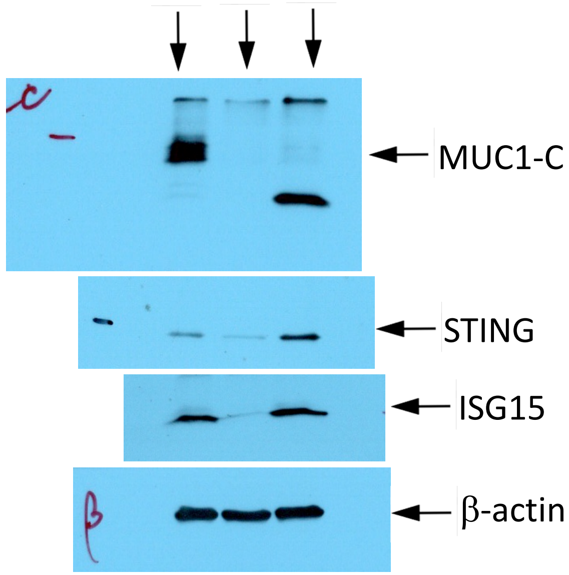

Figure S5A

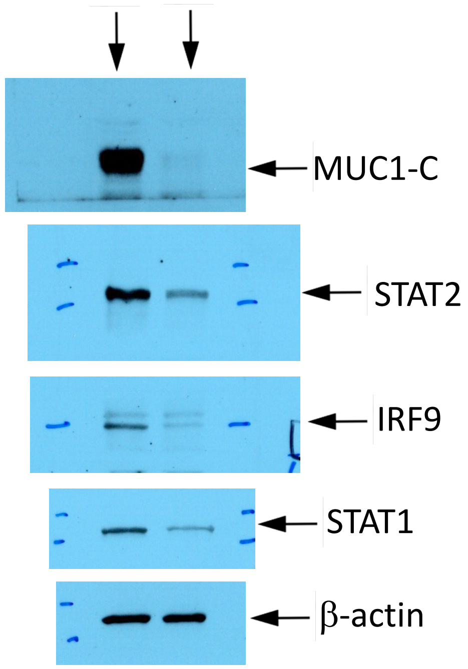

Figure S7A

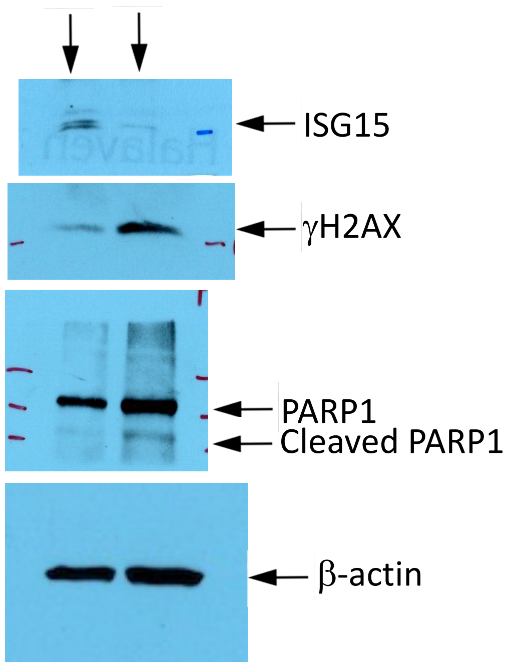

Figure S7B

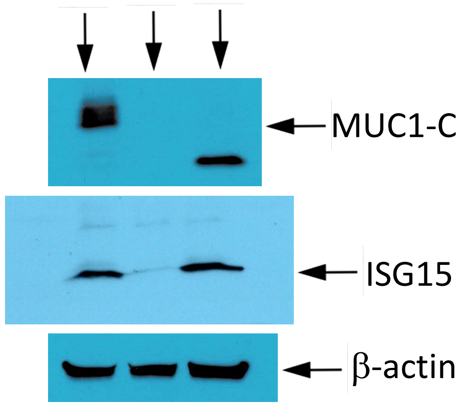

Figure S7C

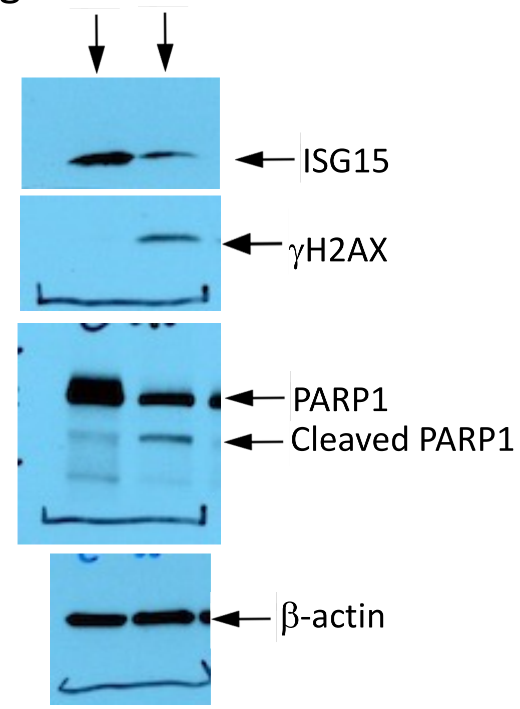

Supplement: Supplementary file 1 [file cancers-14-02580-s001.zip › cancers-1687207 original images.pdf]
