# Supplementary material for: Targeting MUC1-C Suppresses Chronic Activation of Cytosolic Nucleotide Receptors and STING in Triple-Negative Breast Cancer
Source: Cancers (Basel). 2022 May 24;14(11):2580. doi: 10.3390/cancers14112580 (PMC9179855; doi:10.3390/cancers14112580)
Supplement: Supplementary file 1 [file cancers-14-02580-s001.zip › cancers-1687207 supplementary.pdf]

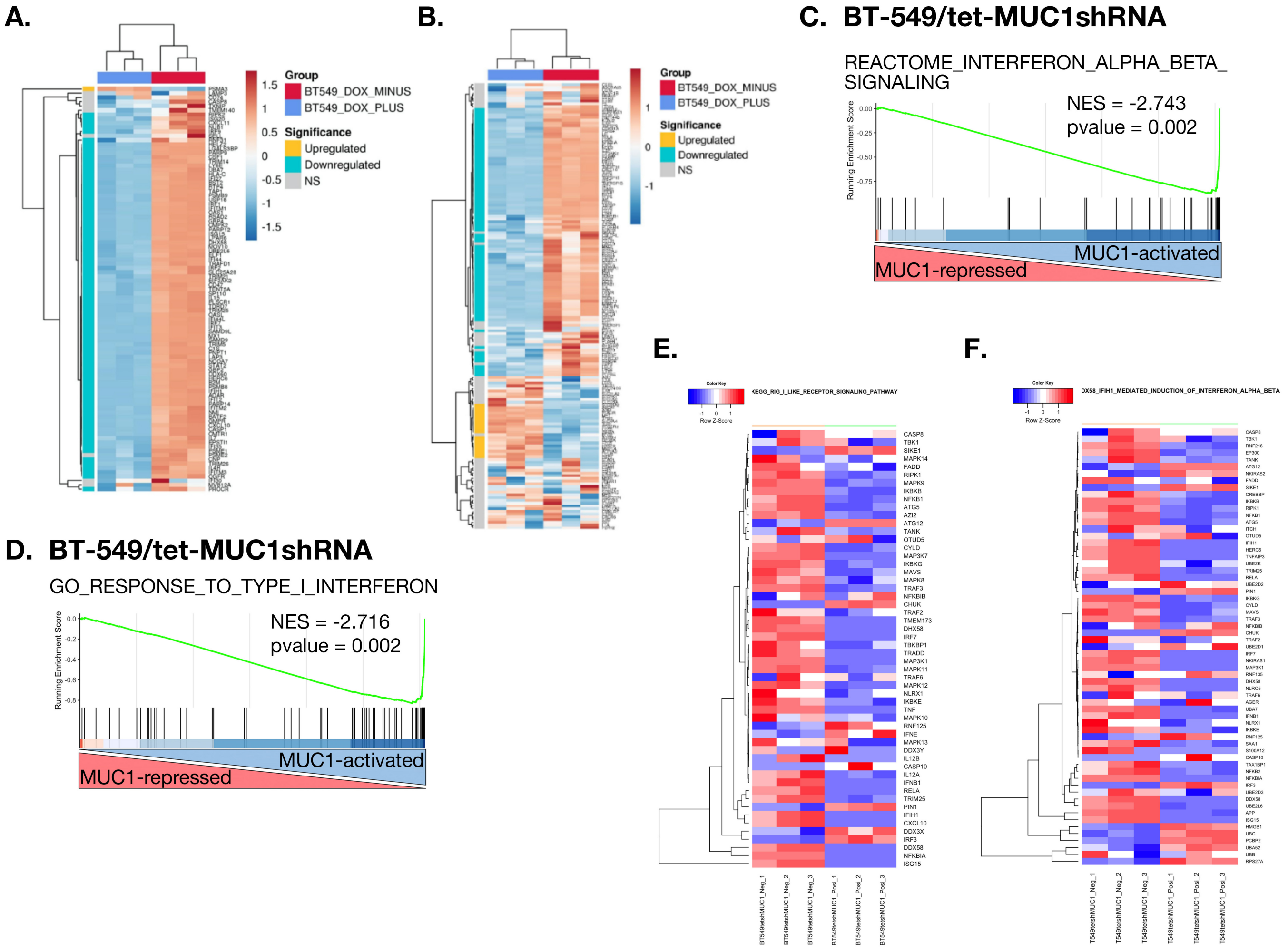

Supplemental Figure S1. Effects of silencing MUC1-C on expression type I IFN pathway genes. RNA-seq was performed in triplicate on BT-549/tet-MUC1shRNA cells treated with vehicle of DOX for 7 days. **A and B.** Heatmaps comparing the effects of MUC1 silencing on genes from the HALLMARK INTERFERON ALPHA RESPONSE (**A**) and HALLMARK INFLAMMATORY RESPONSE (**B**) pathways. **C and D.** Candidate pathway enrichment plots for the REACTOME INTERFERON ALPHA BETA SIGNALING (**C**) and GO RESPONSE TO TYPE I INTERFERON (**D**) pathways. **E and F.** Heatmaps comparing the effects of MUC1 silencing on genes from the KEGG RIG-I LIKE RECEPTOR SIGNALING REACTOME (**E**) and DDX58 IFIH1 MEDIATED INDUCTION OF INTERFERON ALPHA BETA (**F**) pathways.

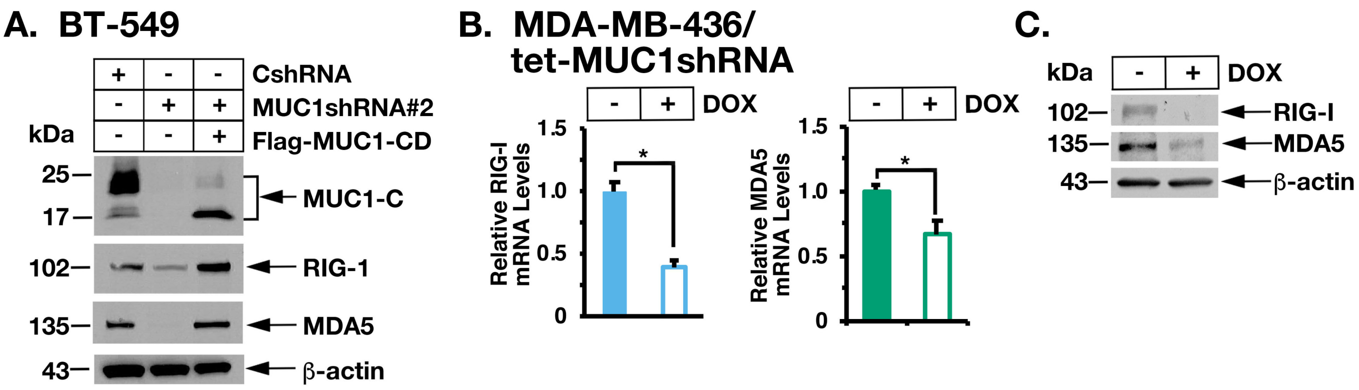

Supplemental Figure. S2. Silencing MUC1-C in TNBC cells suppresses RIG-I and MDA5 expression. A. Lysates from BT-549 cells expressing CshRNA, MUC1shRNA#2 and Flag-MUC1-CD were immunoblotted with antibodies against the indicated proteins. **B and C.** MDA-MB-436/tet-MUC1shRNA were treated with vehicle or DOX for 7 days. RIG-I and MDA5 mRNA levels were analyzed by qRT-PCR (**B**). The results (mean ± SD of 4 determinations) are expressed as relative mRNA levels compared to that obtained for vehicle-treated cells (assigned a value of 1). Lysates were immunoblotted with antibodies against the indicated proteins (**C**).

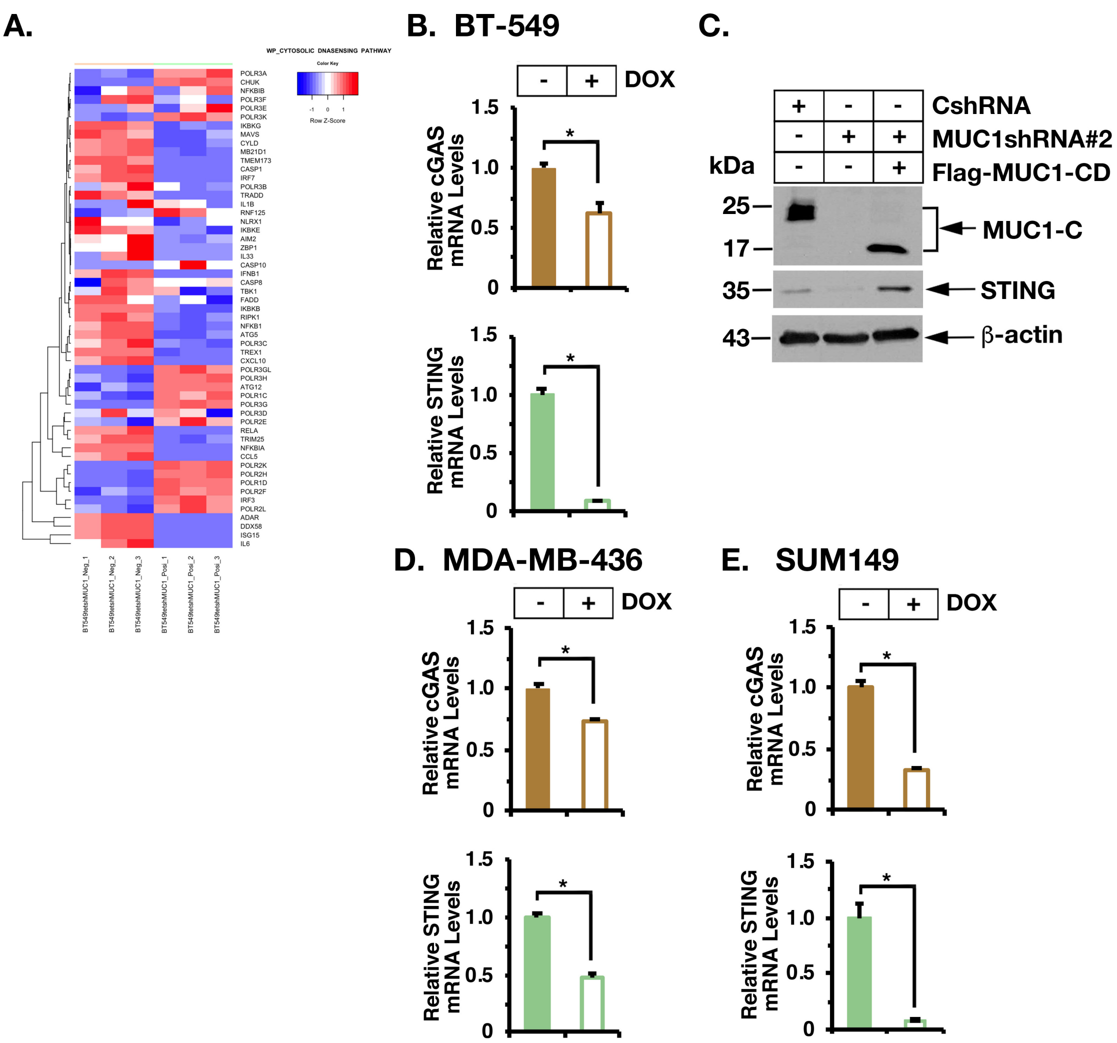

Supplemental Figure S3. MUC1-C induces cGAS and STING expression in BT-549, MDA-MB-436 and SUM149 TNBC cells. A. Heatmap comparing the effects of MUC1 silencing in BT-549 cells on genes from the WP CYTOSOLIC DNA SENSING PATHWAY signature. **B.** BT-549/tet-MUC1shRNA cells treated with vehicle or DOX for 7 days were analyzed for cGAS and STING mRNA levels by qRT-PCR. The results (mean±SD of 4 determinations) are expressed as relative mRNA levels compared to that obtained for vehicle-treated cells (assigned a value of 1). **C.** Lysates from BT-549 cells expressing CshRNA, MUC1shRNA#2 and Flag-MUC1-CD were immunoblotted with antibodies against the indicated proteins. **D and E.** MDA-MD-436/tet-MUC1shRNA (**D**) and SUM149/tet-MUC1shRNA (**E**) cells treated with vehicle or DOX for 7 days were analyzed for cGAS and STING mRNA levels by qRT-PCR. The results (mean±SD of 4 determinations) are expressed as relative mRNA levels compared to that obtained for vehicle-treated cells (assigned a value of 1).

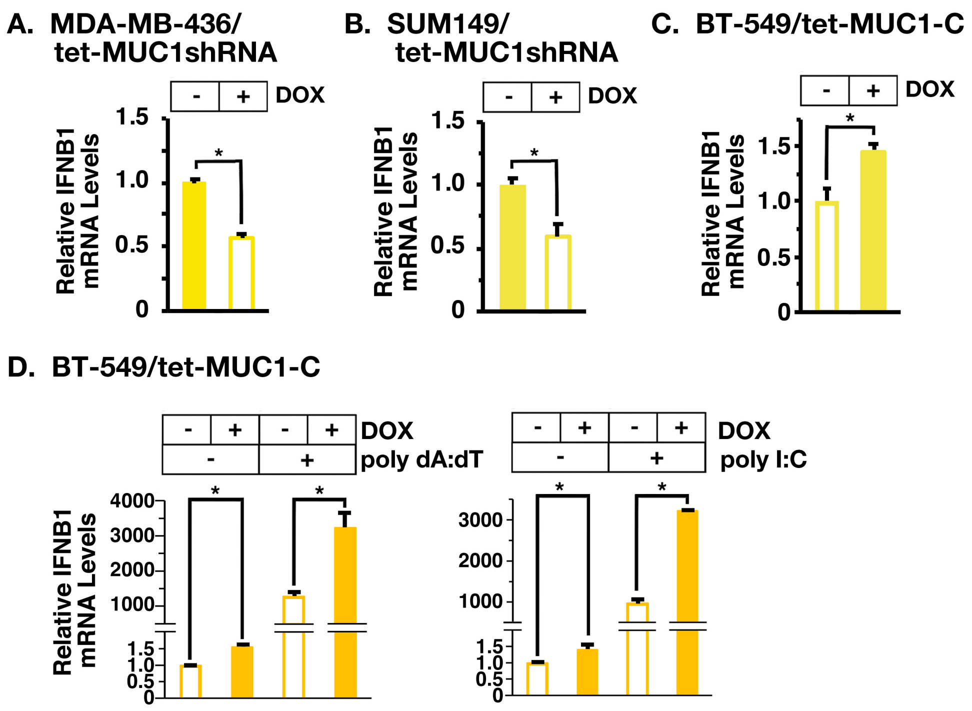

Supplemental Figure S4. MUC1-C drives IFNB1 expression in TNBC cells. A and B. MDA-MB-436/tet-MUC1shRNA (**A**) and SUM149/tet-MUC1shRNA (**B**) cells treated with vehicle or DOX for 7 days were analyzed for IFNB1 mRNA levels by qRT-PCR. **C.** BT-549 cells expressing a tet-inducible MUC1-C vector treated with vehicle or DOX for 7 days were analyzed for IFNB1 mRNA levels by qRT-PCR. **D.** BT-549 cells expressing a tet-inducible MUC1-C vector treated with vehicle or DOX for 7 days were transfected with poly I:C (left) or poly dA:dT (right) for 4 hours and analyzed for IFNB1 mRNA levels by qRT-PCR. The results (mean±SD of 4 determinations) are expressed as relative mRNA levels compared to that obtained for vehicle-treated cells (assigned a value of 1).

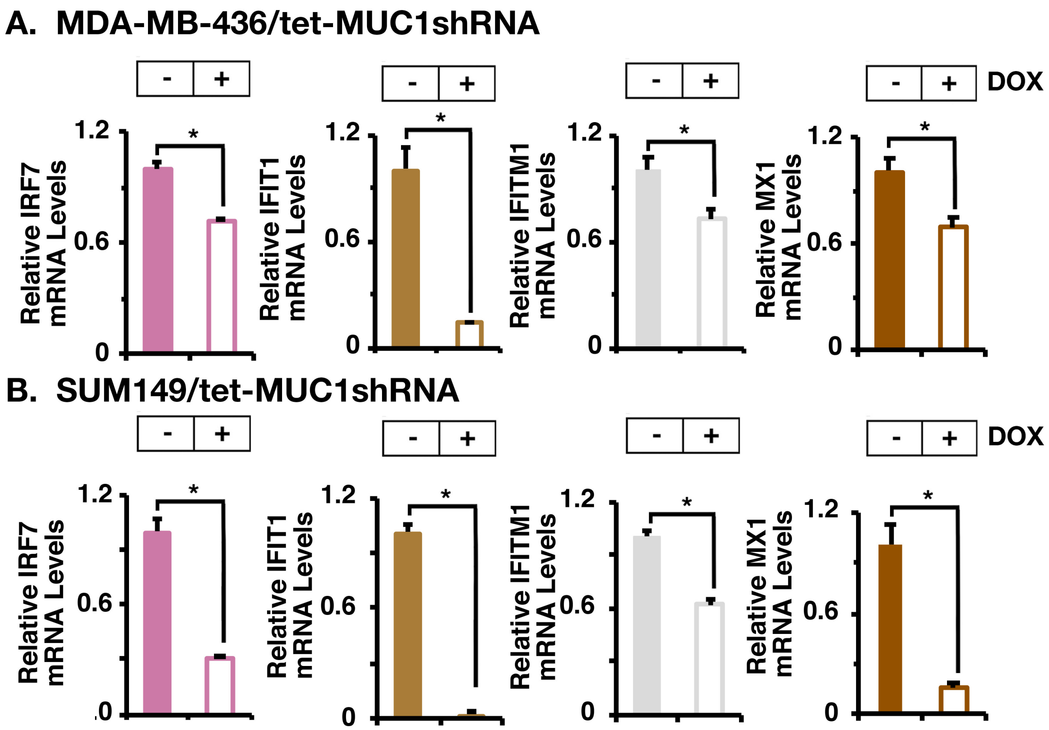

Supplemental Figure S5. Effects of silencing MUC1-C on gene expression. A and B. MDA-MB-436/tet-MUC1shRNA (**A**) and SUM149/tet-MUC1shRNA (**B**) cells treated with vehicle of DOX for 7 days were analyzed for the indicated mRNA levels by qRT-PCR. The results (mean±SD of 4 determinations) are expressed as relative mRNA levels compared to that obtained for vehicle-treated cells (assigned a value of 1).

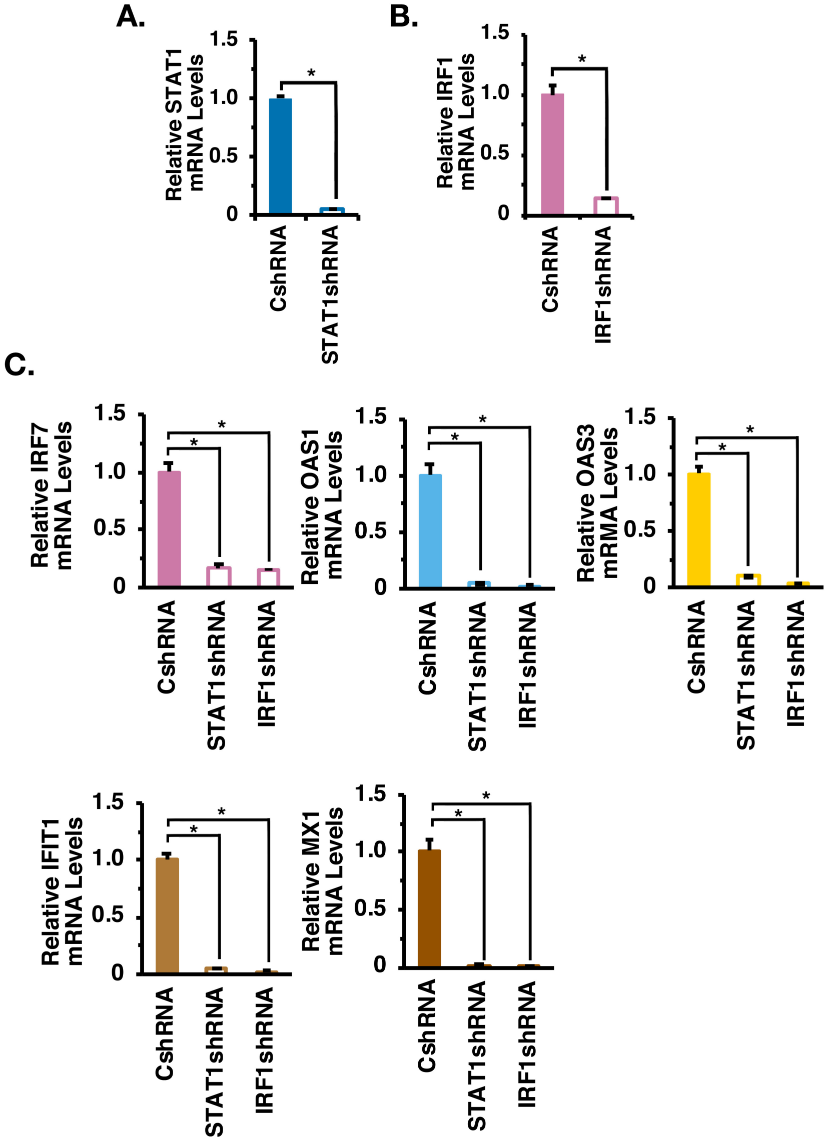

Supplemental Figure S6. Effects of silencing STAT1 and IRF1 on IRDS gene expression. A and B. BT-549 cells expressing a control CshRNA, STAT1shRNA (**A**) or IRF1shRNA (**B**) were analyzed for the indicated mRNA levels by qRT-PCR. **C.** BT-549 cells expressing a CshRNA, STAT1shRNA or IRF1shRNA were analyzed for the indicated mRNA levels by qRT-PCR. The results (mean±SD of 4 determinations) are expressed as relative mRNA levels compared to that obtained for vehicle-treated cells (assigned a value of 1).

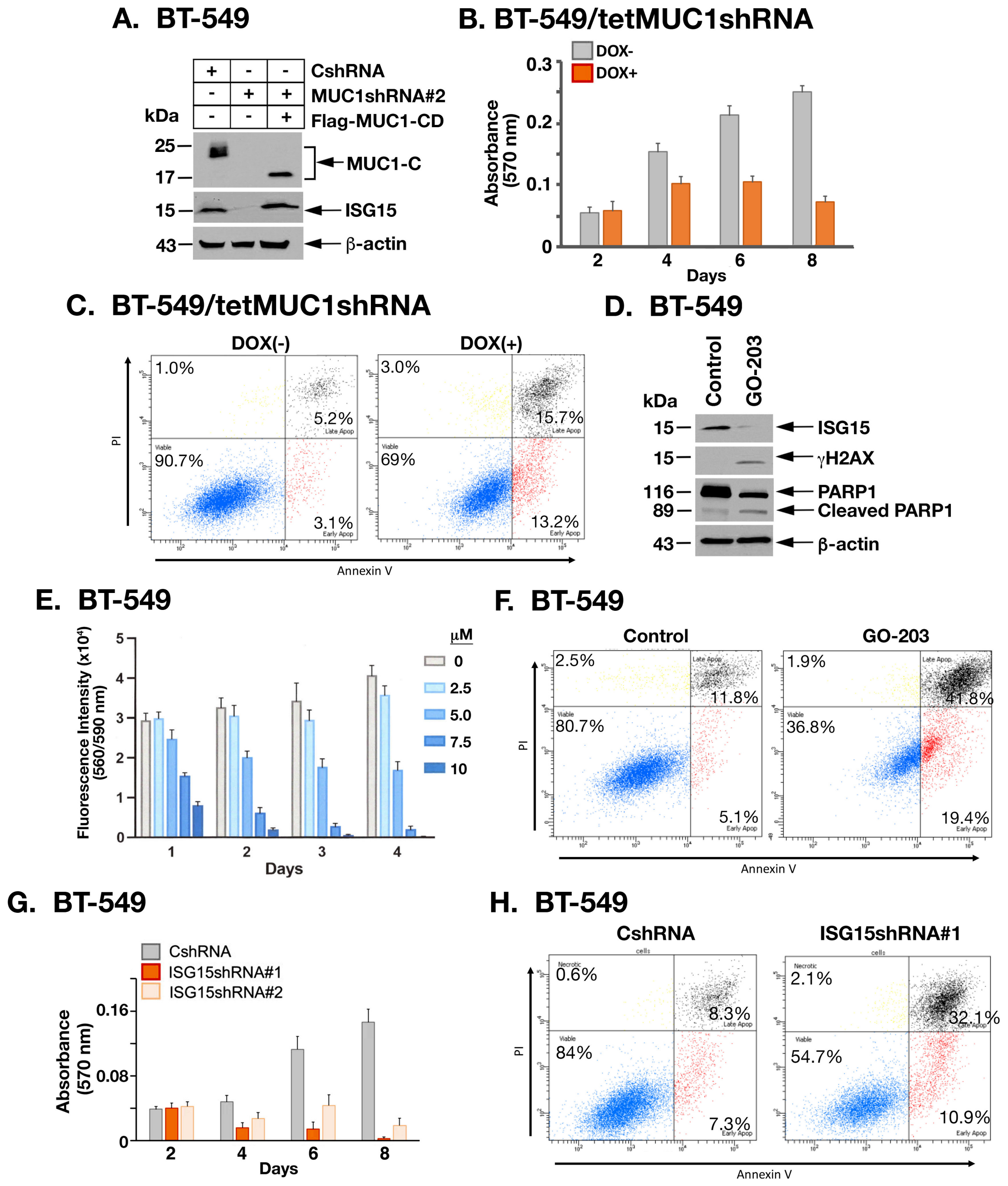

Supplemental Figure S7. Effects of silencing MUC1-C and ISG15 on induction of DNA damage and apoptosis. A. Lysates from BT-549 cells expressing CshRNA, MUC1shRNA#2 and Flag-MUC1-CD were immunoblotted with antibodies against the indicated proteins. **B and C.** BT-549/tet-MUC1shRNA cells treated with vehicle or DOX were monitored at the indicated times after seeding for cell proliferation as determined by the AlamarBlue assay (**B**). Cells were analyzed by flow cytometry for Annexin V and propidium iodide staining at 5 days (**C**). The results are representative of 3 determinations. **D.** Lysates from BT-549 cells left untreated or treated with 10 µM GO-203 for 24 hours were immunoblotted with antibodies against the indicated proteins. **E and F.** BT-549 cells left untreated or treated with the indicated concentrations of GO-203 were monitored at the indicated times for cell proliferation as determined by the AlamarBlue assay (**E**). Cells were analyzed by flow cytometry for Annexin V and propidium iodide staining at 2 days (**F**). The results are representative of 3 determinations. **G and H.** BT-549 cells expressing a CshRNA, ISG15shRNA#1 or ISG15shRNA#2 were monitored at the indicated times after seeding for cell proliferation as determined by the AlamarBlue assay (**G**). Cells were analyzed by flow - cytometry for Annexin V and propidium iodide staining at 3.5 days (**H**). The results are representative of 3 determinations.

Table S1. Primers used for qRT-PCR.

Primer	FWD	REV
**MUC1-C**	AGACGTCAGCGTGAGTGATG	GCCAAGGCAATGAGATAGAC
**STAT1**	GGAACTTGATGGCCCTAAAGGA	ACAGAGCCCACTATCCGAGACA
**STAT2**	GCAGCACAATTTGCGGAA	ACAGGTGTTTCGAGAACTGGC
**IRF1**	CATGGCTGGGACATCAACAA	TTGTATCGGCCTGTGTGAATG
**IRF7**	GCAGCGTGAGGGTGTGTCTT	GCTCCATAAGGAAGCACTCGAT
**IRF9**	CCCGACCTCACCGATGAC	TCTCGCGAAGCTGGATGTC
**RIG-I**	CTGGACCCTACCTACATCCTG	GGCATCCAAAAAGCCACGG
**MDA5**	CCATGGAGAAGGCTGGGG	CAAAGTTGTCATGGATGACC
**CGAS**	TAACCCTGGCTTTGGAATCAAAA	TGGGTACAAGGTAAAATGGCTTT
**STING**	AGCATTACAACAACCTGCTACG	GTTGGGGTCAGCCATACTCAG
**IFNB1**	ATGACCAACAAGTGTCTCCTCC	GGAATCCAAGCAAGTTGTAGCTC
**OAS1**	TGAGGTCCAGGCTCCACGCT	GCAGGTCGGTGCACTCCTCG
**OAS3**	CCGAACTGTCCTGGGCCTGATCC	CCCATTCCCCAGGTCCCATGTGG
**IFIT1**	TTGCAGGAAACACCCACTTCT	GCAAAGCCCTATCTGGTGATG
**MX1**	CTTTCCAGTCCAGCTCGGCA	AGCTGCTGGCCGTACGTCTG
**IFITM1**	GGATTTCGGCTTGTCCCGAG	CCATGTGGAAGGGAGGGCTC
**ISG15**	CGCAGATCACCCAGAAGATCG	TTCGTCGCATTTGTCCACCA
**β-actin**	GATGAGATTGGCATGGCTTT	CACCTTCACCGTTCCAGTTT
**GAPDH**	CCATGGAGAAGGCTGGGG	CAAAGTTGTCATGGATGACC

Table S2. Primers used for ChIP-qPCR.

Primer	FWD	REV
**pRIG1**	GGAGGGAAACGAAACTAGCC	TTAAAGCCGGGTAGGAGGAG
**pMDA5**	CTTTGTAAACGTAATCTGCCTGG	GCTTTCCTTTTCTGTTTCCCG
**pSTING**	GTGCAGCCTGAAAATGAGATG	TTCCTACCTCCCTTCCTGAG
**pISG15**	CGGTTTTGTTTCTTCCGCTCA	AGCACCGGCCCTATTATAAGC
